# The economic costs of orthopaedic services: a health system cost analysis of tertiary hospitals in a low-income country

**DOI:** 10.1186/s13561-024-00485-8

**Published:** 2024-02-17

**Authors:** Pakwanja Twea, David Watkins, Ole Frithjof Norheim, Boston Munthali, Sven Young, Levison Chiwaula, Gerald Manthalu, Dominic Nkhoma, Peter Hangoma

**Affiliations:** 1 University of Bergen, Bergen, Norway; 2 Ministry of Health, Lilongwe, Malawi; 3 University of Washington, Seattle, WA USA; 4 Lilongwe Institute of Orthopaedics and Neurosurgery, Lilongwe, Malawi; 5 University of Malawi, Zomba, Malawi; 6 Kamuzu University of Health Sciences, Blantyre, Malawi; 7 Chr. Michelson Institute (CMI), Bergen, Norway; 8 University of Zambia, Lusaka, Zambia

**Keywords:** Orthopaedic trauma, Traumatic injuries, Time driven activity based costing, Top-down approach, Tertiary level hospitals

## Abstract

**Background:**

Traumatic injuries are rising globally, disproportionately affecting low- and middle-income countries, constituting 88% of the burden of surgically treatable conditions. While contributing to the highest burden, LMICs also have the least availability of resources to address this growing burden effectively. Studies on the cost-of-service provision in these settings have concentrated on the most common traumatic injuries, leaving an evidence gap on other traumatic injuries. This study aimed to address the gap in understanding the cost of orthopaedic services in low-income settings by conducting a comprehensive costing analysis in two tertiary-level hospitals in Malawi.

**Methods:**

We used a mixed costing methodology, utilising both Top-Down and Time-Driven Activity-Based Costing approaches. Data on resource utilisation, personnel costs, medicines, supplies, capital costs, laboratory costs, radiology service costs, and overhead costs were collected for one year, from July 2021 to June 2022. We conducted a retrospective review of all the available patient files for the period under review. Assumptions on the intensity of service use were based on utilisation patterns observed in patient records. All costs were expressed in 2021 United States Dollars.

**Results:**

We conducted a review of 2,372 patient files, 72% of which were male. The median length of stay for all patients was 9.5 days (8–11). The mean weighted cost of treatment across the entire pathway varied, ranging from $195 ($136—$235) for Supracondylar Fractures to $711 ($389—$931) for Proximal Ulna Fractures. The main cost components were personnel (30%) and medicines and supplies (23%). Within diagnosis-specific costs, the length of stay was the most significant cost driver, contributing to the substantial disparity in treatment costs between the two hospitals.

**Conclusion:**

This study underscores the critical role of orthopaedic care in LMICs and the need for context-specific cost data. It highlights the variation in cost drivers and resource utilisation patterns between hospitals, emphasising the importance of tailored healthcare planning and resource allocation approaches. Understanding the costs of surgical interventions in LMICs can inform policy decisions and improve access to essential orthopaedic services, potentially reducing the disease burden associated with trauma-related injuries. We recommend that future studies focus on evaluating the cost-effectiveness of orthopaedic interventions, particularly those that have not been analysed within the existing literature.

## Introduction

Surgically treatable traumatic injuries pose a significant disease burden, causing higher mortality rates than HIV/AIDS, Malaria, and Tuberculosis combined [1] and contributing to 11% of the global disease burden, 88% of which is injury/trauma related [2, 3]. Evidence shows that 90% of deaths from traumatic injuries occur in low- and middle-income countries [4], but the countries face substantial service gaps. Two billion people globally cannot access essential surgery [5, 6]. When access is available, it is often inequitable, favouring high-income countries [1, 2].

Compared to other regions, Sub-Saharan Africa has the highest burden of potentially preventable disability-adjusted life years (DALYs) from injuries, with most orthopaedic trauma cases arising from Road Traffic Injuries (RTIs) [2, 7–9]. It is estimated that RTIs cause approximately 25% of all injuries, making it the eighth-leading cause of mortality and the sixteenth-leading cause of disability globally [1]. Within Sub-Saharan Africa, Malawi has one of the highest road traffic mortality rates at 31 per 100,000 people [10, 11]. Recent studies in Malawi estimate a prevalence of musculoskeletal impairments of 6.5% and 9.5% for children of all ages, respectively [12, 13].

Barriers to accessing general and orthopaedic surgical services have been highlighted on both the supply and demand sides. On the supply side, there is deficient capacity and insufficient investment in strengthening surgical systems, particularly regarding human resources, equipment, and information systems [14–17]. On the demand side, cultural – patients’ beliefs and financial barriers also limit access to surgery [17, 18]. In Malawi, healthcare access problems exist due to geographical, financial, and cultural reasons [19]. Despite the low investment in surgical capacity in LMICs, including Malawi, surgery is highly cost-effective [20] and has the potential for significant economic benefit, mainly because surgically treatable conditions are more prevalent among the younger and more productive members of society [2, 3].

Even though most orthopaedic trauma cases occur in low and middle-income countries, most economic evaluation literature on this subject is concentrated in high-income countries [21–23]. Ali et al. [23] noted the scarcity and poor quality of economic evaluation studies in low-income countries, highlighting the research disparity in orthopaedic trauma literature, with most studies focusing on common fractures like femur fractures, neglecting the broader spectrum of diagnoses in these regions. In addition, the disparity in the costs of delivering orthopaedic care between low- and high-income countries, as Schade et al. [21] reported, makes applying results from high-income settings to low-income settings challenging. Furthermore, resource requirements and costs vary based on context and change over time. Therefore, there is a critical gap for more comprehensive information regarding the costs and effectiveness of surgical interventions in low and middle-income countries [14, 23, 24]. Filling this evidence gap will have planning and policy implications in LMIC health systems [24].

Our study aimed to provide context-specific evidence on the cost of orthopaedic services in low-income settings. Our approach was to estimate tertiary level-of-care specific costs and the cost of care by diagnosis in two Malawian tertiary-level hospitals. To our knowledge, no previous study has aimed to estimate the total orthopaedic costs at the hospital level and the diagnosis-specific costs of multiple orthopaedic interventions in low-income countries.

## Methodology

### Study setting

Malawi, situated in sub-Saharan Africa, is characterised as a low-income country, with a GDP per capita of $511 in 2021 [25], and according to the 2020 NHA report, a per capita spending on health amounting to $39.8 [26]. The healthcare system in Malawi is structured into three levels: primary, secondary, and tertiary. At the tertiary level, four hospitals offer general and specialised medical services: Kamuzu Central Hospital, Queen Elizabeth Central Hospital, Mzuzu Central Hospital, and Mzuzu Central Hospital. Healthcare services, including orthopaedic services, are delivered through public health facilities, private-not-for-profit facilities, and private for-profit facilities. Notably, public and private-not-for-profit health facilities are the primary providers of healthcare services within the national health system [27]. Public health services are predominantly free in public facilities, except for optional paying services. Voluntary health insurance schemes pool about 4.1% of the total health expenditure, while out-of-pocket payments account for 12.6% [26].

We conducted this costing study in two purposively sampled facilities: Kamuzu Central Hospital (KCH) and Mzuzu Central Hospital (MCH), located in the central and northern parts of the country. KCH was chosen as the location for a new specialised orthopaedic hospital, serving as a reference point for future costing studies. In contrast, MCH was selected as a smaller comparator hospital. Both hospitals are in urban areas and offer general, speciality, teaching, and research services. KCH is a 1200-bed facility and sees over 120,000 outpatients and 35,000 patients annually. In contrast, MCH is a 300-bed facility and sees over 90,000 outpatients and 19,000 inpatients annually.

The study took a health system perspective and used a mixed costing methodology. We estimated the costs per outpatient, per inpatient by diagnosis, and the annual cost of orthopaedic services. A top-down approach was used to estimate the direct and indirect economic costs attributable to the orthopaedic department. In contrast, the Time-Driven Activity-Based Costing (TDABC) approach was used to estimate the diagnosis-specific costs. Two primary considerations informed the selection of diagnoses for inclusion in this study. Firstly, we focused on the number of recorded cases, ensuring an adequate number of patients to observe treatment heterogeneity and identify patterns that could inform and confirm assumptions about the treatment pathway. Secondly, given the comparative nature of our study between two hospitals, the chosen diagnoses needed to be prevalent in both facilities to facilitate meaningful cost comparisons.

We collected retrospective cost and epidemiological data for one year, from July 2021 to June 2022. From the retrospective review of patient files, we obtained information on diagnoses, prescription patterns, diagnostic tests, surgeries, and other treatments done on the patient. After adjusting for inflation, we recorded the costs in Malawi Kwacha and converted them to United States Dollars. The price reference year used for this study is 2021.

### Costing process

#### Top-down approach

We followed the process Shepard et al. [28] recommended for the top-down costing approach. First, we identified and classified the cost centres within the hospital. We identified three cost centre types—direct cost centres, intermediate cost centres, and indirect cost centres. Similarly, we classified the inputs as direct or indirect based on their relation to the cost unit. We then estimated the total cost of each input, assigned the unit costs to cost centres, and then allocated all the costs to the final cost centres. The total costs were the sum of all inputs.

### Resource item measurement and valuation

#### Personnel costs

Each hospital’s human resource department provided information on the number of staff by cadre. We used the Government salary scale to calculate the costs for each cadre. We excluded donor payments to staff due to a lack of data. We calculated the direct staff costs as the full-time equivalent based on time allocated to orthopaedic service delivery. Interviews with management staff informed staff allocations to the orthopaedic department. We allocated the indirect human resource costs to orthopaedic services based on service utilisation relative to all other services at the hospital.

#### Medicines and supplies

To obtain the total costs of medicines and supplies, we reviewed pharmacy requisition records of all direct and indirect cost centres providing services to orthopaedic patients. The department's total cost was then calculated as the product of the volume and price for all the items. The unit costs for medicines and supplies were derived from the Central Medical Stores catalogue and supplemented by hospital procurement records. We apportioned costs to the orthopaedic department for the administrative cost centres based on service utilisation.

#### Overhead and other administrative costs

We obtained financial expenditure data from the hospital finance department.

#### Capital costs

We collected information on the type and number of medical equipment in the orthopaedic service delivery areas from the hospital asset register and direct observation. We used recent procurement records and supplier catalogues to obtain the current unit costs and estimated equipment lifetimes. For buildings, we physically measured the floor area for each building in the hospital and used valuations by the Government Buildings Department to estimate replacement costs. We then calculated the Equivalent Annual Cost of Capital (EAC) using the formula in the appendix using a discount rate of 3%. Equipment and building lifetimes were based on literature recommendations on expected useful life years for each equipment type.

#### Intermediate output costs

We obtained data on the output volume from each hospital's laboratory and radiography departments and input utilisation data from pharmacy requisition records for the two departments. Output data was obtained from the hospital administration and medical case records. We apportioned costs to the orthopaedic department based on service volume and expert opinion.

Details on the expenditure items and apportionment criteria are provided in Table 1 below.

**Table 1:** Summary of cost components and assumptions for the top-down costing

Cost Component	Data Source	Unit Price Data Source	Allocation Basis	Apportioning Statistics	Data Sources
Personnel	Hospital Human Resource Records	Government Salary Scale	Workstation within the hospital Patient volume (diagnostic and administrative staff)	Number of Patients	[29]
				Length of Stay	
				Procedure duration	
				FTE^a^	
Medicines and Consumables	Hospital Pharmacy requisition records	Central Medical Stores Trust catalogue	100% allocation (exclusively orthopaedic patient areas)	Number of Patients	[29, 30]
			Based on service utilisation (joint use areas)	Number of Patients	
Overheads – electricity, water, security, cleaning	Hospital Accounting Records	Finance Department	Floor area	Floor Area	[29, 31]
Other administrative Costs	Hospital Accounting Records	Finance Department	Based on service utilisation	Number of patients	[31, 32]
Medical Equipment	Hospital Asset Register	Procurement Records	100% allocation (exclusively orthopaedic patient areas)	Number of Patients	[29]
	Physical Count	Procurement Agency Catalogues	Based on service utilisation (joint use areas)		
Buildings	Direct measurement	Valuation from the Department of Buildings	100% allocation (exclusively orthopaedic patient areas)	Number of Patients	[29, 33]
			Based on service utilisation (joint use areas)		

^a^Full time equivalent

### Time-driven activity-based costing approach

We followed the process Rubin [34] recommended to estimate the diagnosis-specific costs by documenting the clinical management pathway– a sequence of tasks that are part of the treatment for each diagnosis—based on interviews with healthcare workers and patterns observed during the patient file review. For every diagnosis, the inputs and the duration of each procedure were based on the information provided by health workers. We then listed, for each task, the inputs required, and the time taken to complete the task. To account for heterogeneity in input use across patients, we used the actual utilisation patterns observed in the medical records to estimate the treated fraction for each input.

For hospital personnel, we estimated the cost rate per minute. The available working minutes were calculated after adjusting for public holidays and paid time off and then divided the cost by the available working minutes. For capital, we calculated the cost rate per minute after adjusting for equipment idle time (based on the department and working hours) and assumed an equipment downtime rate of 20% [35]. The cost per patient per non-consumable input was calculated as the cost rate multiplied by the time required for the resource. The cost per diagnosis is the sum of the inputs for all the activities. We included the following aspects of the treatment in the micro-costing: patient evaluation on admission, diagnostics tests, surgical procedures, blood transfusion, physiotherapy, and hospital stay. We did not include surgery complications and post-discharge costs.

The total overhead costs were allocated to the orthopaedic department based on the cost drivers in Table 1 above. We calculated the per-patient costs for inpatients and outpatients assuming one inpatient day: three outpatient visits equivalence scale and calculated patient-day equivalents using the formula in Appendix 1 [36, 37]. To account for non-task-specific human resource costs for inpatients, we adopted the approach by Diab et al. [38], calculating the personnel cost per inpatient day based on staff Full-Time Equivalents allocated to the orthopaedic department. Total per-person personnel and overhead costs were estimated by multiplying the overhead per patient day and the personnel cost per patient day by the diagnosis-specific average length of stay. We also calculated the weighted mean costs for each diagnosis using the number of patients as the weighting factor. The Activity Based Costing approach and assumptions are documented in Table 2 below.

**Table 2:** Summary of cost components and assumption for the micro-costing

Cost Component	Costing Methods	Assumptions for the intensity of need	Missing data protocol	Precedence
Medicines	Mean prescribed medication based on patient records	The proportion of patients prescribed medication	Records with missing prescription doses are excluded from consideration	[30, 31]
	The cost is calculated per item based on pack size		The items missing the pack size used the most commonly available or efficient pack size	
	Total cost = cost per item *dose* frequency per day * Duration			
Consumables	Average usage per person	Volume based on expert opinion	NA^b^	[31]
Personnel	Task-specific cost – cost per minute * task duration	Task duration – expert opinion	NA	[31, 38, 39]
	Inpatient costs – daily personnel cost per ward/ average # of inpatients per day	Adjusted FTEs^c^		
Diagnostic Tests / Blood Transfusion	Based on average utilisation for specific diagnoses and expert opinion	The proportion of patients utilising service and expert opinion	NA	
Medical Equipment	Calculated cost per use	Orthopaedic-specific use/utilisation	NA	[29, 31, 33, 37]
Overheads	Department apportioned overhead costs from top-down costing	1-bed day: 3 outpatient visits	NA	[31, 34, 40]

^b^Not applicable

^c^Full Time Equivalent

To capture the normative cost, we assumed, based on interviews with orthopaedic specialists, that 100% of fracture patients in the study period initially had a cast applied on admission to the orthopaedic ward based on health worker accounts, even though this was not always indicated in the patient files. We also assumed all fracture patients had x-rays done, even though this was only sometimes specified in the patient files. By doing this, we consider the disparity between the actual practice and the treatment norms. To make comparisons between the two hospitals, we used the F test of significance.

### Addressing uncertainty

We conducted a one-way sensitivity analysis to assess uncertainty in our study. The variables examined included the discount rate, given the varying recommendations in costing studies, and logistical costs not explicitly identifiable in hospital expenses, usually covered by the Central Government. Although not explicitly identified in hospital costs, supply chain costs were included in the analysis due to their potential contribution to total costs. We varied the discount rate between 3 and 5%, following recommendations for higher rates in low- and middle-income countries [41]. Additionally, we explored supply chain costs ranging from 0 to 20%, considering estimates from Sarley et al. [42] that ranged from 1 to 44% based on the product and variables included in the cost analysis. We created alternative scenarios incorporating these adjustments.

## Results

### Patient profiles across the hospitals

The overall number of patients included in the study was 2,372 (Table 3). Our analysis only included patients whose patient files were made available to the research team. In the case of KCH, the number of files reviewed was less than the reported total cases, implying missing data (14%). In the case of MCH, the included patients were more than the recorded number of cases (20%). All patients who were admitted and whose files were available for review from 1st June 2021 to 31st July 2022 were included in the study. Most admitted patients were male (71%) and in the 20-to-59 age range (55%). The mean length of stay (LOS) ranged from 1 to 67 days depending on the diagnosis, with the highest average LOS from Tri-malleolar fractures.

**Table 3:** Patient summary statistics

Patient Characteristics	MCH^d^(*n* = 964)	KCH^e^(*n* = 1420)	*p*-value^1^
Gender			
Male	636 (67%)	1068 (75%)	
Female	316 (33%)	352 (25%)	
Age Group			
Under 1	0	6	
1 to 5	72 (7%)	101 (7%)	
6 to 19	277 (29%)	353 (25%)	
20 to 59	498 (52%)	824 (58%)	
60 above	105 (11%)	136 (9%)	
LOS			
Average LOS^f^(Orthopaedics)	10	12	0.000
Median	8	11	
Low–high	(1–49.5)	(1–67)	
Average LOS (Femur Fracture)	20	19	
Cause of Injury			
Road Traffic Accidents	209 (22%)	497 (35%)	
Other Causes	743 (78%)	923 (65%)	
Other Statistics			
Number of orthopaedic beds	58	228	
Number of Orthopaedic Surgeons	1	4	
Readmission Rate	8%	3%	

*P* ≤ 0.05 was considered to indicate statistical significance

^d^Mzuzu Central Hospital

^e^Kamuzu Central Hospital

^f^Length of Stay

### Total department costs and cost composition

**Fig. 1:**
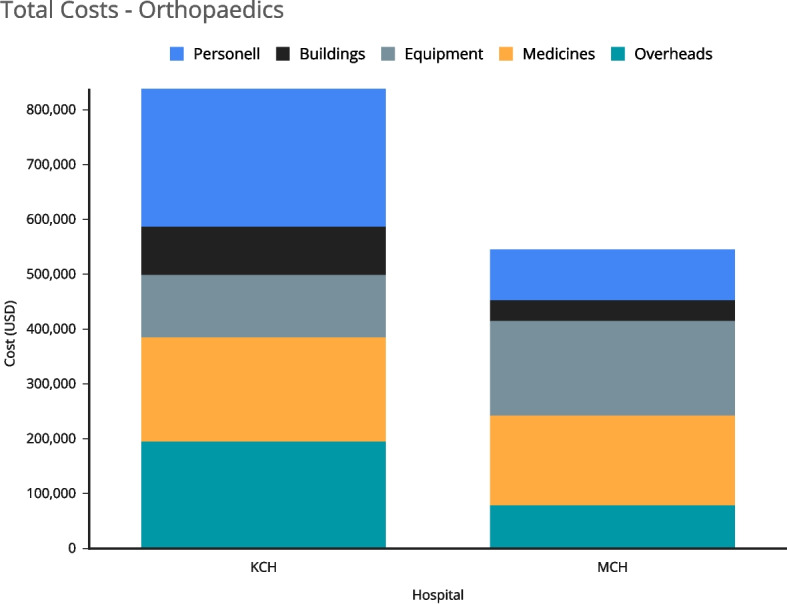
Total costs illustrates the estimated annual costs of the orthopaedic departments of Mzuzu Central Hospital (MCH) and Kamuzu Central Hospital (KCH). Unit costs by Diagnosis and Service Area

The estimated annual costs of the orthopaedic department at MCH were $ 545,254 and $838,540 for KCH (Fig. 1). The cost per inpatient day at KCH was $43 compared to $53 at MCH, while the cost per outpatient visit was $14 at KCH compared to $18 at MCH.

Medicines and consumables accounted for the highest total costs at MCH (37%), while personnel accounted for the highest total costs at KCH (48%). The difference in the contribution of personnel costs total costs was due to comparatively more specialised and non-specialised staff at KCH compared to MCH. The direct service delivery-related costs at both hospitals accounted for the highest proportion of total costs (66% at MCH and 77% at KCH).

**Fig. 2:**
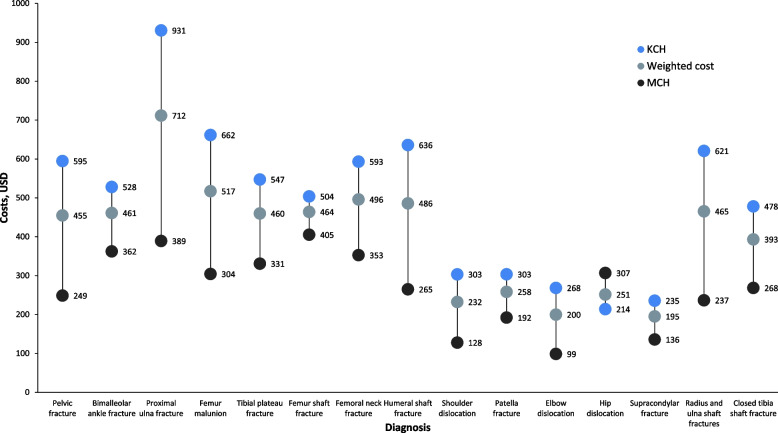
Mean Costs by Diagnosis provides a succinct overview of the weighted mean costs categorized by diagnosis. Notably, Kamuzu Central Hospital (KCH) exhibits higher mean treatment costs by diagnosis than Mzuzu Central Hospital (MCH)

Figure 2 below provides summaries of the weighted mean costs and total mean costs, categorized by cost item and service provided, as well as by diagnosis and hospital. Among the diagnoses, proximal ulna fractures incurred the highest weighted mean costs at $714, while supracondylar fractures had the lowest weighted mean costs at $195. The biggest cost drivers were personnel, drugs, consumables, and overheads. The mean treatment costs by diagnosis were higher for KCH than MCH due to comparatively longer average lengths of stays, relatively longer waiting times for surgery, higher surgery rates, and a higher staff-to-patient ratio in the admission wards. A comparison of costs by intervention and service area shows that the most significant contributors to total costs were inpatient days, which accounts for staff time and overhead costs per patient day and operating room costs, including personnel time, drugs and consumables, and equipment costs. The diagnostic and imaging costs were the lowest contributor to total costs and the least likely to vary across diagnoses.

### Sensitivity analyses

**Fig. 3:**
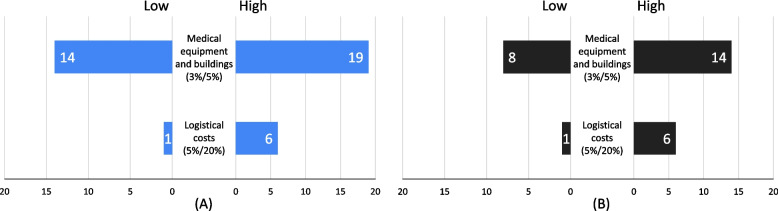
Sensitivity Analysis. presents the sensitivity analysis results conducted for Kamuzu Central Hospital (**A**) and Mzuzu Central Hospital (**B**). The analysis focuses on the impact of logical costs and discount rates on the total. The findings show that both hospitals exhibit a higher sensitivity to changes in discount rates compared to logical costs

Figure 3 presents the results of the one-way sensitivity analysis for both hospitals. In both hospitals, the change in total costs because of varying the logistical costs from 5 to 20% is within a similar range at 1% at MCH and 1% at KCH. In comparison, at 20% logistical costs, the change in total costs rises to 6% at both hospitals. Total costs are more sensitive to changes in the discount rate from 3 to 5%, increasing the total costs from 8 to 13% at MCH and from 13 to 19% at KCH.

## Discussion

Considering the increasing burden of surgically treatable conditions in low- and middle-income countries and the lack of context-specific data, our study aimed to estimate the total and the diagnosis and intervention-specific costs associated with delivering orthopaedic services in tertiary hospitals in Malawi. We used top-down costing methods to estimate the total costs of orthopaedic services in two tertiary-level hospitals and time-driven activity-based costing to estimate the costs by diagnosis. We constructed a patient pathway based on health worker interviews and utilisation patterns observed in the patient files. The TDABC costing methodology allowed us to estimate the costs of each activity that is part of the treatment pathway while adjusting for the intensity of need based on actual utilisation patterns.

In low-income countries, there is a dearth of orthopaedic costing studies. When available, they often focus on single diagnoses and compare treatment methods like traction and intramedullary nailing, primarily for severe fractures such as femur and tibia. For femur fracture treatment costs, our study aligns with findings from Dar es Salaam [43] at $418 (MCH) and $512 (KCH) versus $530.87. A previous study in Malawi [38] reported costs of $597 (intramedullary nailing) and $678 (traction) for femur shaft fractures. A Cambodian study [44] estimated per-patient costs of $826 for intramedullary nailing, primarily due to more extended stays. We found personnel and overhead costs to be the primary cost drivers, consistent with other low-income countries. A study in Tanzania [45] reported mean treatment costs of $426 for the Intramedullary Nailing group and $559 for the external fixation group compared to $331 (MCH) and $547 (KCH) for tibia fractures, aligning with our findings and suggesting their applicability in similar settings.

The average length of stay (LOS) was 10.2 days at MCH and 12.4 days at KCH, comparable to a study in Tanzania [7] with an estimated LOS of 11 days. For femur fractures, the LOS at MCH and KCH (20 days and 19 days, respectively) contrasts with eight weeks in Sierra Leone and 45 days in Ethiopia [46, 47]. The total estimated orthopaedic service delivery costs were $556,924 at MCH and $838, $540 at KCH, with costs per inpatient day being $53 and $43, respectively.

Compared to the costs associated with treating other prevalent diseases in low-income countries, orthopaedic interventions tend to incur slightly higher costs. The estimated costs of tuberculosis treatment range from $258 to $315.30 per individual [48, 49]. The annual per person cost of undergoing Anti-retroviral treatment for HIV is approximately $792 [50] In comparison, selected maternal health interventions exhibit varied cost ranges: Antenatal Care spans from $7.24 to $31.42, normal delivery ranges from $14.32 to $278.22, and caesarean delivery fluctuates from $72.11 to $378.94 [51]. However, in practical terms, healthcare professionals consider various factors beyond costs when prioritizing healthcare interventions, including cost-effectiveness, equity, disease burden, and budget impact.

Our study revealed variations in average treatment costs between the two hospitals, primarily driven by differences in skill mix and length of stay. Both hospitals face human resource shortages below Malawi government standards, which, if addressed, could enhance outcomes. Skill mix and length of stay significantly contribute to overall treatment costs. While KCH has lower costs per bed-day and outpatient visit, MCH consistently demonstrates lower diagnosis-specific treatment costs. This discrepancy can be attributed to extended hospital stays, longer surgery waiting times, more complex trauma cases, and a higher patient volume. This trend is particularly evident at KCH, where there is lower variability in average treatment costs due to extended stays.

To our knowledge, this is the first study to estimate the diagnosis-level costs of multiple orthopaedic interventions in the same paper. These cost estimates are beneficial to provide indications of the cost-of-service delivery outside of the most common orthopaedic conditions that are usually costed in the literature. Based on a comprehensive review of patient files over one year, we attempted to value the service delivery inputs for selected diagnoses according to diagnosis and estimate average costs per diagnosis.

There were some limitations to our study. Our estimates are based on the primary diagnosis and do not include treatment complications, multiple fractures, co-morbidities, or post-discharge costs. As the patient-level data were aggregated before analysis, we did not isolate cases with treatment complications or record co-morbidities. While we understand that post-discharge costs, particularly for rehabilitation, can contribute to total treatment costs, we could not include these costs due to a lack of data. Our assumptions of the intensity of the need for treatment inputs are based on observed utilisation patterns and are, therefore, different for each hospital. The costs could be underestimated due to missing data for some patients who received treatment during the period under review. The selection of diagnoses for inclusion in the study was determined by the available data and an assumption of completeness of the patient files.

Another limitation of our study is that we did not incorporate the time to surgery into our cost calculation. In situations where waiting times for surgery are prolonged, this factor could contribute to the overall cost of treatment, and we recommend that this is considered in subsequent costing studies. Given that the duration of stay is widely recognised as a significant factor influencing costs, the generalizability of our results may be restricted, and any extension to other settings should be approached with caution. Our analysis also did not include the cost of non-medical furniture and tools, as well as allowances provided by donors. In practical terms, donor contributions to personnel costs are not routine and often vary by specific tasks, making them unpredictable and non-uniform. However, when data is accessible, it is considered best practice to include these allowances in the overall costing to ensure a more accurate reflection of the actual cost of service delivery.

The use of a mixed costing methodology introduces another limitation, as it limits the comparability of the results with other settings where different costing methodologies have been used. On the TDABC, we aimed to calculate the normative cost of service delivery while accounting for current treatment practices. This involved making assumptions about the coverage of tasks done as part of the treatment process even if the tasks are not done for every patient. Consequently, our results should be interpreted as normative costs, and the implications of these assumptions should be considered when interpreting the findings. We propose that future research studies should address the cost-effectiveness of orthopaedic diagnoses that have received comparatively less attention in the existing literature.

## Conclusion

Using gross and micro-costing methods, we estimated the total costs of delivering orthopaedic services at two tertiary hospitals in Malawi. Ours study finds that there are disparities in the average treatment costs which are primarily driven by differences in length of stay, treatment patterns, and skill mix. Our study was designed to fill a gap in the literature on the costs of providing orthopaedic services in a low-income country and inform decision-making, particularly around the cost of service provision for orthopaedic interventions.

## Appendices

1. Human Resource Capacity per Minute

$$HR\;Cost\;Rate=\frac{Annual\;Salary}{Full-time\;annual\;capacity\;in\;minutes}$$

2. Equivalent Annual Cost of Capital

$$EAC=Asset\;Price\times\frac r{1-\left(1+r\right)^{-t}}$$

where: $$\text{r is the discount rate}$$

$$\text{t is the number of useful life years for each type of equipment}$$

3. Equipment Cost Rate

$$Medical\;equipment\;Cost\;Rate=\frac{EAC}{Annual\;capacity\;in\;minutes}$$

4. Patient Day Equivalents and Overhead Cost per Unit

$${PDE}_{outpatient}=\left(annual\;in\;patient\;days\times\frac1{Weighing\;Factor}\right)+annual\;Outpatient\;Visits$$

$${PDE}_{inpatient}=\left(annual\;Outpatient\;Visits\times Weighing\;Factor\right)+annual\;in\;patient\;days$$

$$Overhead\;cost\;per\;outpatient\;visit=\frac{annual\;overhead\;expenditure}{{PDE}_{outpatient}}$$

$$Overhead\;cost\;per\;Inpatient\;Day=\frac{annual\;overhead\;expenditure}{{PDE}_{inpatient}}$$

## Data Availability

The datasets generated and analysed during the current study are not publicly available due to restrictions imposed by the ethics approval granted for this research and institutional policies prohibiting the public sharing of sensitive or confidential data. Access to the data may be granted upon request and subject to prior approval by the ethics committee and institutions involved.
