# Correction: The economic costs of orthopaedic services: a health system cost analysis of tertiary hospitals in a low-income country

**DOI:** 10.1186/s13561-024-00511-9

**Published:** 2024-06-15

**Authors:** Pakwanja Twea, David Watkins, Ole Frithjof Norheim, Boston Munthali, Sven Young, Levison Chiwaula, Gerald Manthalu, Dominic Nkhoma, Peter Hangoma

**Affiliations:** 1 University of Bergen, Bergen, Norway; 2 Ministry of Health, Lilongwe, Malawi; 3 University of Washington, Seattle, WA USA; 4 Lilongwe Institute of Orthopaedics and Neurosurgery, Lilongwe, Malawi; 5 University of Malawi, Zomba, Malawi; 6 Kamuzu University of Health Sciences, Blantyre, Malawi; 7 Chr. Michelson Institute (CMI), Bergen, Norway; 8 University of Zambia, Lusaka, Zambia

**Correction:** ***Health Econ Rev*** **14, 13 (2024)**

10.1186/s13561-024-00485-8

Following publication of the original article [1], the authors corrected a mistake in the affiliation of the second author David Watkins, it was erroneously stated, “University of Washington, Washington, DC, USA” instead of the correct affiliation, “University of Washington, Seattle, WA, USA”.

The affiliation has been updated above and the original article [1] has been corrected.
